# Study protocol, increasing awareness and early detection in students at risk of gynaecological cancer: feasibility of an online educational and behaviour change intervention

**DOI:** 10.1186/s40814-026-01801-1

**Published:** 2026-04-21

**Authors:** Patricia Holch, Anne-Marie Bagnall, Seren Hughes, Lauren K. McSorley

**Affiliations:** 1 Department of Psychology, School of Humanities & Social Sciences, Leeds Beckett University, Portland Building, Room PD 403 A, City Campus, Leeds, LS1 9HE UK; 2 School of Health, Leeds Beckett University, 10th floor Calverley Building, City Campus, Leeds, LS1 3HE UK

## Abstract

**Background:**

Around 2500 teenagers and young adults (TYA) in the UK are diagnosed with cancer each year, many with melanomas or carcinomas. Almost a third are diagnosed via A&E, highlighting the need for earlier detection. Although university students recognise common cancer symptoms, they often miss less obvious ones (e.g. constipation, frequent urination, bloating) and may delay seeking help. Early exposure to cancer information during key life stages, such as university can promote lifelong body awareness and earlier help-seeking. We have created an online behaviour-change intervention for university students, including a gynaecological cancer awareness film and a volitional help sheet to support intention to seek help. The aim is to assess its feasibility and acceptability, explore barriers and enablers to engagement, and refine the intervention ahead of a full UK university rollout.

**Methods:**

We will conduct a 1:1 randomised longitudinal mixed-methods feasibility study with 86 eligible UK university participants (women or anyone at risk of gynaecological cancer, aged 18+). Participants will complete an online gynaecological cancer educational film and/or a volitional help sheet via Qualtrics™. Cancer awareness (YPCAM) and Theory of Planned Behaviour measures will be collected at baseline, 4 weeks, 3 months, and 6 months. A subset of the intervention group will take part in semi-structured interviews on barriers and enablers to uptake. Feasibility will be assessed through recruitment and attrition rates, adherence, and study completion. Mean score changes between groups across timepoints will be analysed, adjusting for baseline scores. Interview data will be analysed using reflexive thematic analysis. Progression to a full trial requires achieving at least 80% of the recruitment target.

**Discussion:**

Findings from this study will show whether the intervention can improve cancer awareness and help-seeking for gynaecological symptoms among TYA. The long-term aim is to use these insights to support wider adoption of cancer awareness initiatives in universities. Providing cancer information early in students’ lives may help them establish lasting habits around body awareness and encourage them to share this knowledge with others.

**Trial registration:**

Clinicaltrials.gov from 21/08/2025 https://clinicaltrials.gov/study/NCT07147283.

**Supplementary Information:**

The online version contains supplementary material available at 10.1186/s40814-026-01801-1.

## Background

More than 2500 young people are diagnosed with cancer each year in the UK and while cancer in young people is varied, the majority diagnosed are either melanomas or other carcinomas (including gynaecological cancers) [1]. Alarmingly, almost a third (29%) of teenage and young adult (TYA) cancers in the UK are diagnosed in accident and emergency departments [2]. Early diagnosis is key to improved cancer outcomes [3]. Indeed, improving early detection of cancer in young people is one of the 10 priority areas for the James Lind Alliance Priority Setting Partnerships [4]. Importantly, late presentation of gynaecological cancers in particular has a detrimental effect on patient outcomes [5–7].

Gynaecological cancers affect both younger and older women (or trans or non-binary individuals with female anatomy), cervical being the most prevalent followed by ovarian, uterine, and vulval [8].

However, in preliminary work with university students, although they could identify the common cancer warning signs (e.g. lumps, bleeding), they could not identify those less common including constipation, frequency in urination, and bloating [9, 10]. Further, despite awareness of warning signs, most young people would delay or avoid seeking help altogether, citing barriers to help seeking including embarrassment or wasting health professionals’ time [11].

The key to lifelong monitoring and early detection of cancer in young people is the early exposure to cancer information and body awareness at a formative age [12]. Moreover, the years at university are seen as a significant transitional life stage facilitating students to establish positive health behaviours [13].

### Rationale for the proposed study

With a view to developing a cancer educational intervention for current university students, we conducted a series of Public and Patient Involvement and Experience (PPIE) consultations with 50 psychology students at LBU. During these sessions, we used the principles of co-design [14] to discuss the priorities required from young people for an online educational and behavioural intervention to promote early detection and diagnosis of cancer. The priorities identified included that the intervention should be online, delivered by personnel who are knowledgeable (and if possible, with lived experience), and to be delivered in ‘bite size pieces’ and have an ‘active’ component where students would be required to think. In this current study, we have used these principles to develop an online intervention to support the early detection and intention to seek help for symptoms of gynaecological cancer. The intervention is based around the signs and symptoms of gynaecological cancers (e.g. abnormal bleeding, bloating, abdominal pain, frequency in urination/constipation, changes in vulva) [15, 16]. We envisage ultimately that this study (when fully powered) will have (i) immediate impact on cancer awareness and early detection in young people at university, (ii) enable participants to form monitoring and awareness intentions that will be maintained, and (iii) explore whether there will be upward communication of cancer awareness and early detection from students to family members as evidenced previously [17, 18]. However, firstly we need to pilot the components of the whole intervention with university students and understand the barriers and enablers to uptake.

## Aims and objectives

### Study aims

1. To assess the feasibility of the intervention, understand the barriers and enablers of uptake.
2. Generate important data on how to improve the intervention to develop a definitive study for launch in UK universities.

### Primary objectives

To establish the:

- Uptake of the intervention via recruitment (we aim to recruit 80% of our intended sample) and retention rates
- Number of withdrawals from the study, exploring the reasons why where possible to
- Number of times (and for how long) the educational film was viewed
- Time taken to complete the volitional help sheet (to establish engagement)
- Optimum time for follow-up (post intervention)—through recruitment rates

To explore intervention group participants’ views via semi-structured interviews including:

- Acceptability of the intervention and views on how to improve it
- The barriers and enablers to accessing cancer awareness information and for help seeking for symptoms
- Whether there had been upward communication around cancer between TYA and parents and friends
- The likelihood of development and maintenance of cancer monitoring and healthy habits in the future

### Secondary objective(s)

To inform the selection of a primary outcome measure for a definitive future trial, we will assess the performance of the Young Person’s Cancer Awareness Measure (YPCAM) [19] and Theory of Planned Behaviour (TPB) [20] questionnaires at baseline, 4 weeks, 3 months, and 6 months by:

- Determining the extent of floor and ceiling effects from quantitative outcome measures
- Assessing the amount of missing data in quantitative outcome measures
- Assessing the trends in cancer awareness and intention to help-seek between the intervention and control arms (controlling for baseline differences)

## Methods

### Design

**Fig. 1:**
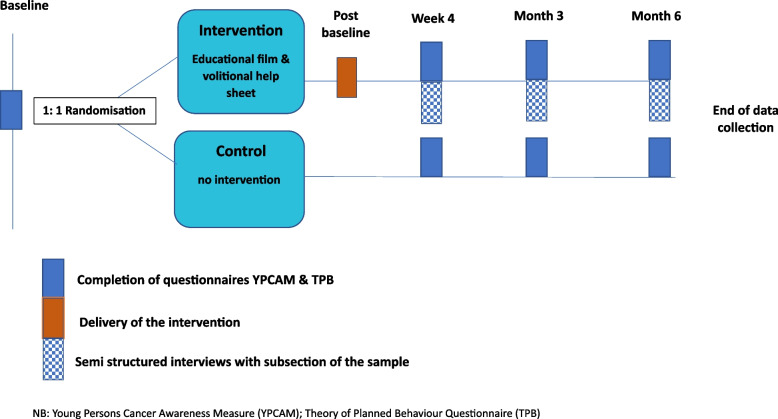
Schematic of study design

This is a randomised mixed-methods feasibility study with repeated measures (see Fig. 1) and adheres to the Medical Research Council (MRC) guidance on the development, implementation, and evaluation of complex health care interventions [21]. Simple randomisation will be established within Qualtrics by assigning random values (e.g. group = 1 or group = 2) to respondents, using branching logic to display the film and volitional help sheet to the assigned intervention group. Young women (or transexual or non-binary individuals with female anatomy) at UK universities will be randomised with 1:1 allocation to either an intervention (an online gynaecological cancer awareness film and volitional help sheet implementation behaviour change intervention) or the control condition via the randomisation facility in Qualtrics™. Participants will be blind to the randomisation sequence via the allocation within Qualtrics; no procedures for unblinding of participants were utilised in this trial as those in the intervention arm were given access to the intervention on completion of the trial.

Cancer awareness and plans for self-monitoring will be assessed at baseline, 4 weeks, 3 months, and 6 months, along with assessment of the theory of planned behaviour domains (Supplementary information 2). Feasibility of the intervention will be assessed by recruitment and retention rates and qualitative interviews. As this was a population of university students, it is clear that many unforeseen circumstances could be out of our control, e.g. exams, deadlines, and holidays, with this in mind students will be reminded to complete their follow-up sessions.

### Participants

Female students (or those at risk of gynaecological cancer, e.g. have ovaries, a uterus, cervix). This may include people who are non-binary, trans masculine/men, women, and/or people who were assigned female at birth attending UK universities. Students will have to be over 18, UK residents, and able to understand written English. Demographic information will be collected such as age, ethnic group, gender, which university they attend, and marital, educational, and employment status which can be fully seen in YPCAM (Supplementary information 1).

### Sample size

We aim to recruit 2 PPIE group members to the study. For the survey, we aim to recruit 43 students per arm (total *n* = 86) as guided by the literature which is sufficient to estimate recruitment as wide confidence intervals are expected [22, 23], and given we are not estimating effect sizes [24] and reflecting a similar sample size of a previous university-based study [13]. We aim to conduct 6 qualitative interviews at each of the three follow-up stages (4 weeks, 3 months, and 6 months) (*n* = 18 in total); these will be selected from the intervention group.

- Female students (or those at risk of gynaecological cancer, e.g. have ovaries, a uterus, cervix)
- Attending UK universities
- UK residents over 18
- Able to understand written and verbal English

Exclusion criteria:

- Those not at risk from gynaecological cancer
- Non-UK residents
- Not a student at a UK university
- Unable to understand written and verbal English
- Under 18 years

### PPIE group

We have a person with lived experience as a grant co-applicant (*n* = 1) and we also aim to recruit a small group of students (*n* = 2) from LBU to guide the project throughout. The PPIE group will be engaged in the development of the interview schedule, participant facing documents, study, and intervention materials. Participants will be reimbursed for their time by using vouchers of monetary value (£20).

### Intervention

The intervention consists of a bespoke online gynaecological cancer awareness educational film and a volitional help sheet to plan future gynaecological help seeking and monitoring based on implementation intention principles [25]. The intervention will be delivered online using Qualtrics™.

### Control

Participants assigned to the control condition will have no exposure to cancer education over and above what is available generally and be assessed on their previously accrued cancer information. They will take part in the study and complete the questionnaires at baseline, 4 weeks, 3 months, and 6 months. To ensure equipoise with the intervention group, control participants will be informed they will have an opportunity to view the educational film and complete the implementation intention volitional help sheet after they have completed the final questionnaire.

### Educational film

**Fig. 2:**
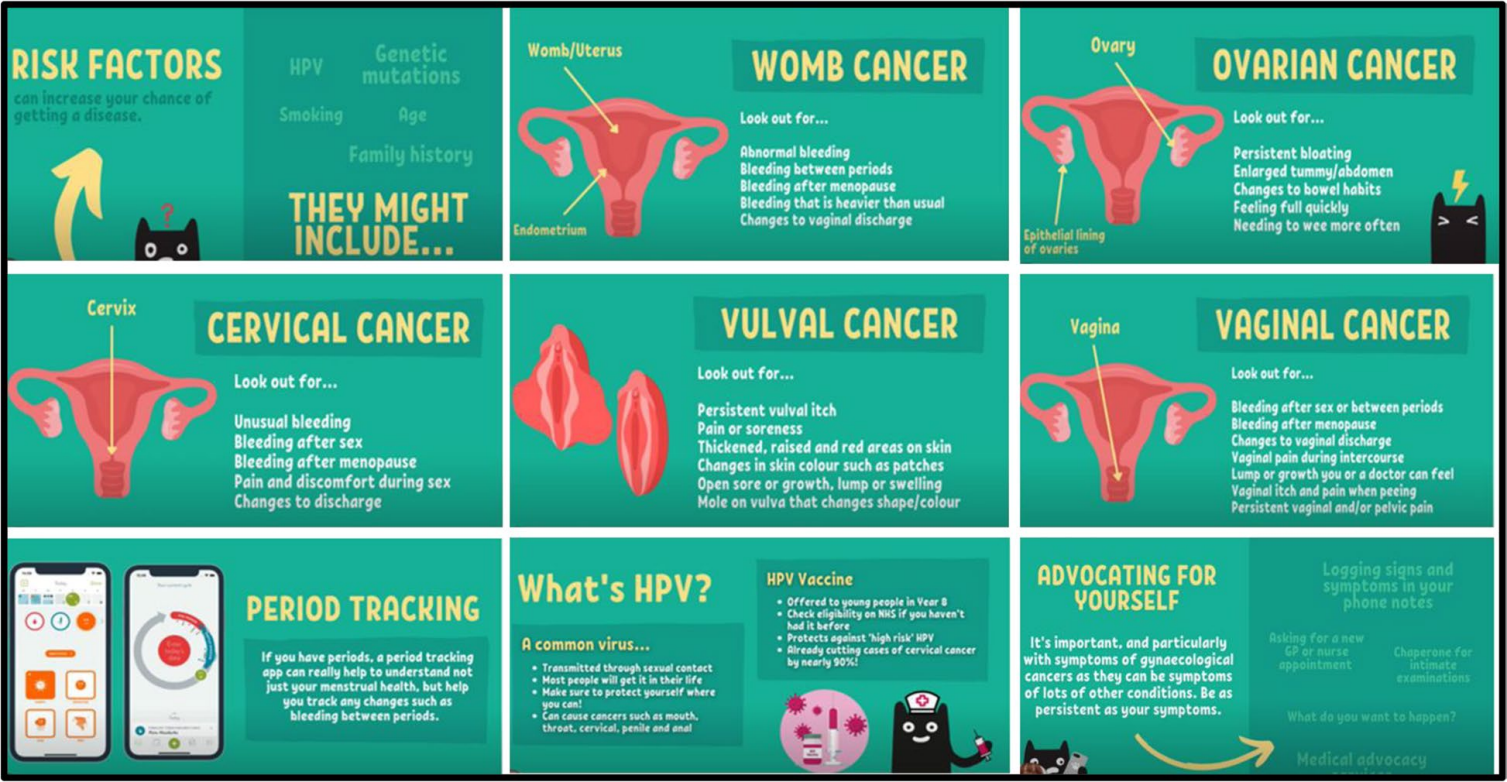
Screenshots from the bespoke educational film on gynaecological cancers commissioned from CATTS

A 20-min educational film has been commissioned from the charity ‘Cancer Awareness in Teens and Twenties’ (who promote cancer awareness, empowerment, and help seeking in young people) outlining the gynaecological cancer warning signs and symptoms (financed by internal LBU University funding). This film provides information about the signs and symptoms of the five different forms of gynaecological cancer: womb cancer (2 min); ovarian cancer (2 min); cervical cancer and cervical screenings (4 min); vulval and vaginal cancer (3 min); human papilloma virus (HPV) and HPV vaccine (3 min); risk factors (2 min), period tracking (2 min), and advocating for themselves (2 min). The film will be delivered online to participants via an online questionnaire builder (QUALTRICS™) accessible via PC, MAC, tablet, and phone. See Fig. 2 for screen shots indicating some of the different areas covered in the film.

### Volitional help sheet

**Fig. 3:**
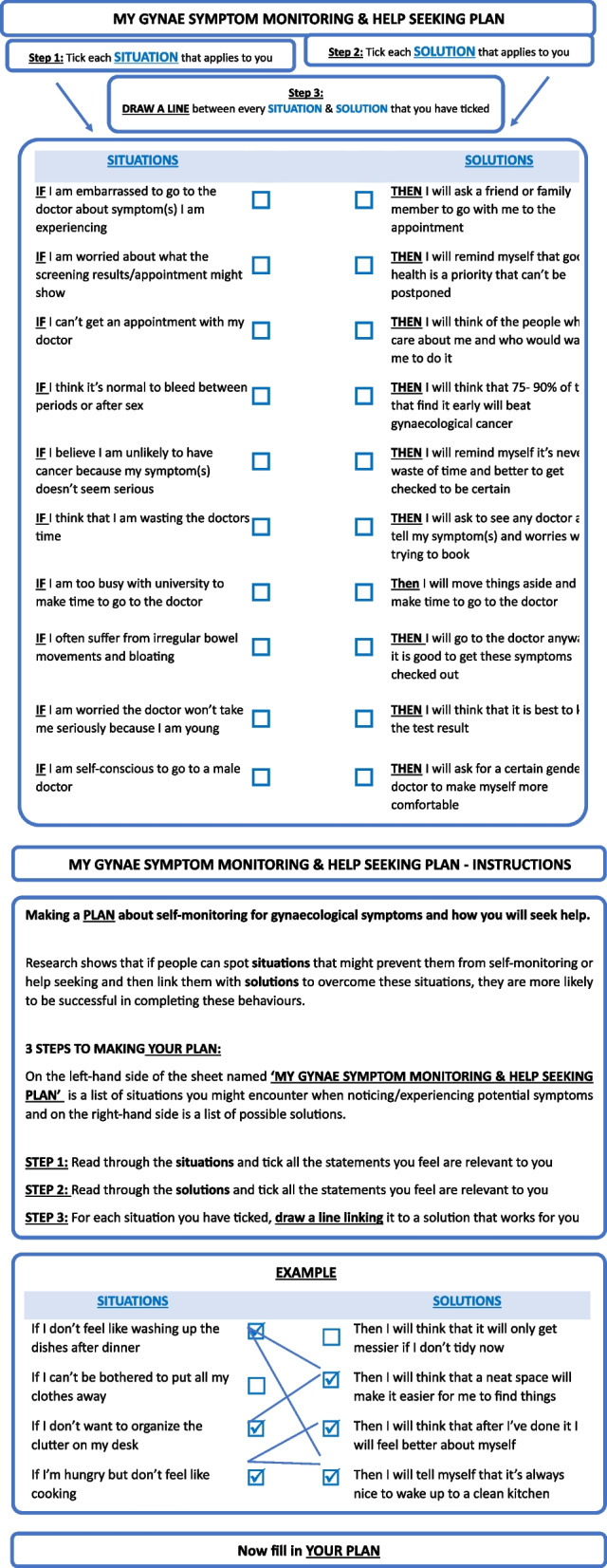
Gynaecology symptom monitoring and help seeking plan

Participants will then take part in an online intervention using implementation intention principles (delivered via QUALTRICS™). Implementation intentions is a robust behavioural technique designed to encourage participants to set tangible goals to self-monitor and help-seek for health problems [26]. Students will self-monitor and plan to act to seek help for potential gynaecological signs and symptoms. *The volitional help sheet encourages participants to self-monitor for gynaecological symptoms and then use a standard self-generation implementation intention to identify cues to help-seeking/action.* The intervention has two components. (1) Self-monitoring: participants will self-monitor for gynaecological symptoms and then use a standard self-generation implementation intention. (2) Cues to help-seeking/action [27]: we will introduce a volitional help sheet which links the cues (of the gynaecological symptoms) with actions and solutions to enable them to seek help (see Fig. 3), e.g. *If I am embarrassed to go to the doctor about symptom(s) I am experiencing, THEN I will ask a friend or family member to go with me to the appointment.*

## Study measures

Young Person’s Cancer Awareness Measure (YPCAM) (Supplementary information 1) at baseline, 4 weeks, 3 months, and 6 months. This 10-item measure is based on the adult Cancer Awareness Measure (CAM) [28] validated in adults (aged ≥18), assessing knowledge of cancer types, incidence rates, warning signs/symptoms, and risk factors, and explores help-seeking intervals, past experience, and potential help-seeking barriers. A further iteration of this instrument (the YPCAM for TYA ≥16) was developed by the Teenage Cancer Trust and Dr Martin McCabe University of Manchester and includes the addition of advocacy and decision-making questions and has been utilised in UK studies [9–11, 29]. However, the YPCAM has not yet been formally validated in TYA and therefore this limits the reliability of the findings.

Theory of Planned Behaviour (TPB) (Supplementary information 2) questionnaire at baseline, 4 weeks, 3 months, and 6 months. The TPB is the most widely used effective model in predicting intention [30, 31], stating that intention is the precursor of behaviour. We have developed a questionnaire incorporating these factors based around a cervical cancer questionnaire used with young women in Ireland [20]. This will explore participants’ attitudes, intentions, subjective norms, anticipated regret, and perceived behavioural control in relation to cancer awareness and help-seeking.

## Materials

### Interview schedule (intervention group only)

A semi-structured interview schedule (Supplementary information 3) was developed to capture views from the intervention group on the acceptability and feasibility of the intervention. The interview explored views on the educational film, the questionnaires, volitional help sheet, and changes they may have made because of the intervention, including potential upward communication to friends and family.

### Qualtrics™

The questionnaire and intervention will be delivered via an online questionnaire builder (Qualtrics™). This is a secure online system recommended by LBU to deliver surveys and questionnaires.

### Microsoft teams

The interviews will be conducted over Microsoft Teams (MS Teams) and the recording and transcription function will be utilised and where appropriate to ensure an accurate transcription this will be checked and enhanced as required by the interviewer as appropriate. This will be completed where possible in a timely manner after the interview was conducted.

## Recruitment, progression criteria, and consent

Participants will be recruited online via a variety of methods:

- Local (LBU) recruitment
  1. Via Leeds Beckett University SONA system (participant recruitment and study management system). A system where students gain participation points for taking part which is beneficial to their degree.
  2. Via posting the study details and Qualtrics™ link to the Leeds Beckett University Student Union Facebook page.
  3. Emailing the study details and Qualtrics™ link to Leeds Beckett University Psychology students via the course email system National (UK) recruitment.
  4. Posting the study details and Qualtrics™ link on Prolific (an online research recruitment platform).
- Progression criterion

The stop-go criteria for progression to a definitive RCT are:

- Green (go)—we aim to recruit ≥ 80% of our intended sample size, i.e. ≥34 in each group.
- Amber (consider proceeding)—a 50%–79% recruitment rate, i.e. 21–33 in each group.
- Red (stop)—not progressing to definitive RCT if recruitment is under 50%, i.e. under 21 in each group.

### Consent

Participants will view an information sheet via Qualtrics™ and if happy to proceed will access and complete the online consent form. The right of participants to refuse consent without giving reasons will be respected. Further, participants will remain free to withdraw from the study up to 2 weeks after taking part without giving reasons by emailing the lead researcher. At this point, their data will also be withdrawn from the study.

## Data handling and storage

### Survey

All anonymised data generated from the survey will be downloaded from Qualtrics into IBM SPSS version 27 for analysis. This will all be stored anonymously on a password-protected University OneDrive account accessible only by the research team.

### Interview data

Interviews will be transcribed verbatim using the recording and transcription function on MS Teams (enhanced by the interviewer where appropriate). Data will be stored in MS Excel/NVIVO/Word documents for analysis. These will all be stored anonymously on the principal investigators (Professor PH) University OneDrive account that is GDPR compliant as recommended by the LBU. No identifiable information will be contained within then stored data.

### Data archiving and data retention

Data will be stored as per LBU guidelines and then deleted. If a participant withdraws consent for their data to be used, it will be deleted immediately.

### Data availability

The data from this study will be available from the authors upon reasonable request. The analysis plan is stored with the LBU ethics committee and the study protocol can be accessed via clinicaltrials.gov.

### Data monitoring

Data will not me monitored via a data monitoring committee given the behavioural nature of the trial, the limited risk to participants, and the small scale of the trial. There are no plans for any interim analyses or guidelines for stopping the study.

### Trial steering committee

The procedures will not be overseen via a trial steering committee as the study is of a small scale and the Trial Management Group have the necessary expertise.

## Planned analyses

### Outcome measures and analysis

As this was a feasibility study, we do not intend to present differences with *p* values as we are not striving for a definitive result, but rather we are interested in size of the effect and the range of plausible values. Therefore, questionnaire data will be analysed descriptively using *n* (%) or mean and standard deviation where appropriate with presentation of 95% confidence intervals.

### Patient adherence to the intervention and study

1. The time taken to watch the educational film and complete the volitional help sheet will be captured via Qualtrics to assess whether the film had been watched and the help sheet had been completed.
2. The total number of questionnaire completions over the four time points over 6 months (24 weeks).
3. Assess attrition rates (to assess the optimum time point for future follow-up on the larger trial).
4. Assess participant withdrawal rates.

### Missing items, floor and ceiling effects, and effects sizes

Missing data items will be examined as the proportion of returned questionnaires with significant number of missing items (as per questionnaires scoring guidance), thus making the calculation of scores not possible.

Score distributions will be examined to detect ceiling and floor effects on questionnaires (defined as >15% of patients reporting highest or lowest scores), by study arm (intervention vs control) and time of data collection. A pooled analysis of all returned questionnaires across all timepoints will also be performed and effect sizes will be determined though sensitivity analyses, where one questionnaire is omitted at a time, and the remaining data is pooled to observe any variations in the results [32].

### Statistical modelling

We will assess the changes in mean scores from participant questionnaires at baseline, to 4 weeks, 12 weeks (3 months), and 24 weeks (6 months) for the intervention and control arms will be calculated. A post hoc exploratory analysis of covariance (ANCOVA) will be performed on the raw scores of completed outcome data for both treatment groups to adjust for a single covariate (baseline scores) [33]. We will present mean differences with 95% CIs (both adjusted and unadjusted) without *p* values, suggested by the CONSORT Statement 2010 extension to pilot and feasibility studies [34].

### Interview data

Data will be analysed using MS Word/Excel file/NVivo and stored on a LBU University OneDrive account as per university guidelines. Interviews will be analysed as per the six stages of reflexive thematic analysis [35–38] and be subject to a hybrid inductive/deductive approach and both semantic and latent coding.

1. Familiarisation: gaining a comprehensive understanding of the interview transcripts.
2. Initial coding: interesting and meaningful codes identified indicating the context of the interview.
3. Searching for themes: interpretative analysis of collated codes, subthemes, and themes.
4. Reviewing themes: deeper review refine/combine/separate themes.
5. Defining and naming themes: providing a unified story.
6. Producing a report: an analytic report using illustrative vivid extracts.

## Modifications

Research ethics committee (REC) will be sought for modifications to the protocol, and any approved changes will be communicated in written form (and reiterated verbally) to all concerned parties including funders, investigators, staff and trial participants, registries, and regulators.

### Details of previous modifications to the protocol where research ethics committee approval was sought

Version 1.0 April 2022, recruitment extended to LBU Facebook pages and advertising through the Psychology BSc and MSc course sites. Version 1.1 August 2022, recruitment open to all levels of student at LBU in addition to a modification to the time points to collect longitudinal data from 6 weeks, 9 months, and 12 months to 4 weeks, 3 months, and 6 months. Version 1.2 March 2023, recruitment extended from LBU to all UK universities to Prolific.

## Dissemination policy

The findings from the study will be disseminated in 6 monthly reports and in a final report (at 24 months) to the Eve Appeal Charity, peer review publications, conferences, and used for educational purposes. Participants will be asked if they agree to this in the consent form.

## Discussion

This paper describes the protocol for the feasibility of an online gynaecological cancer educational and behaviour change intervention in UK female university students (or those at risk of gynaecological cancer). In this mixed-methods study, we aim to determine the feasibility and acceptability of the intervention in young people at UK universities. We will also establish the uptake of the intervention by exploring participant recruitment, retention, and withdrawal rates. We will also examine the process of engagement with the intervention (time taken over viewing the educational film and completing the volitional help sheet). Via interviews, we will investigate the barriers and enablers to uptake of the intervention and will use participants views to refine it for future use. We will also provide evidence of upward communication between young adults and parents and friends around cancer awareness and help seeking and the likelihood of maintenance of cancer monitoring and healthy habits in the future.

The anticipated strengths of our study are that the bespoke educational film is unique in that it is the first gynaecological cancer awareness film internationally developed with young people in mind. Further, the film is paired with the theoretically underpinned implementation intentions (volitional help sheet) which aims to bridge the gap between the ‘intention to’ and actual help seeking behaviours for gynaecological cancer symptoms by using cues to action. However, we acknowledge that having one progression criterion is a limitation of this study design and may not address the multi-dimensional nature of feasibility [39].

We hypothesise that the intervention will be acceptable and useful to participants and that ultimately it will improve their cancer awareness (over time) and willingness to help seek for gynaecological cancer symptoms. Further, we will also gain a clear understanding of the theoretical and behavioural drivers which underpin this behaviour by exploration of the TPB variables.

However, the ultimate success of the intervention is dependent on how well participants engage with the components; here we explore the feasibility and acceptability of the intervention to fully develop and refine it for large-scale testing and roll out.

Ultimately, this intervention has the potential to improve cancer awareness and facilitate help seeking for symptoms. It is clear that and there is a need for universities to actively encourage new health initiatives as an integral part of university life. We hope at this transitional stage of life students will embrace new routines and be open to cancer awareness which will endure over their lifespan.

## Supplementary Information

Supplementary Material 1.

Supplementary Material 2.

## Abbreviations

ANCOVA: Analysis of covariance
CATTs: Cancer Awareness in Teens and Twenties
CONSORT: Consolidated Standards of Reporting Trials
DMP: Data Management Plan
Eve Appeal: Gynaecological charity and funder of the research
HPV: Human papilloma virus
LBU: Leeds Beckett University
MRC: Medical Research Council
NVivo: Qualitative data analysis computer software package
PPIE: Public and Patient Involvement and Experience
PROLIFIC: Participant recruitment platform
Qualtrics™: Online questionnaire builder
SONA: University participant recruitment and study management system
SPSS: Statistical Package for Social Scientists
TBP: Theory of Planned Behaviour
TYA: Teenage and young adult

## References

[1] CRUK. Teenage and young adult (TYA) cancers. https://www.cancerresearchuk.org/about-cancer/childrens-cancer/teenage-young-adult-tya#:~:text=In%20the%20UK%2C%20around%202%2C300,needs%20of%20this%20age%20group.

[2] Teenage Cancer Trust. A third of young people with cancer diagnosed in A&E. 2015. https://www.teenagecancertrust.org/about-us/news/third-young-people-cancer-diagnosed-ae.

[3] Richards MA. The National Awareness and Early Diagnosis Initiative in England: assembling the evidence. Br J Cancer. 2009;101(Suppl 2):S1–4. 10.1038/sj.bjc.6605382. 19956152 10.1038/sj.bjc.6605382 PMC2790704

[4] James Lind Alliance Priority Setting Partnerships. https://www.jla.nihr.ac.uk/priority-setting-partnerships/teenage-and-young-adult-cancer/the-top-10-priorities.htm. Accessed 12/01/2024.

[5] Elias KM, Guo J, Bast RC Jr. Early detection of ovarian cancer. Hematol Oncol Clin North Am. 2018;32(6):903–14. 10.1016/j.hoc.2018.07.003. 30390764 10.1016/j.hoc.2018.07.003 PMC6376972

[6] Ge J, Fader AN, Dudley JC. Early detection of endometrial cancer. Gynecol Oncol. 2023;174:A1–2. 10.1016/j.ygyno.2023.06.010. 37356825 10.1016/j.ygyno.2023.06.010

[7] Kessler TA. Cervical cancer: prevention and early detection. Semin Oncol Nurs. 2017;33(2):172–83. 10.1016/j.soncn.2017.02.005. 28343836 10.1016/j.soncn.2017.02.005

[8] Teenage and young adult cancers (2015–2017). https://www.cancerresearchuk.org/about-cancer/childrens-cancer/teenage-young-adult-tya.

[9] Holch P, Jones G. Vaughan, K, McCabe MG. Cancer awareness, risk perception and barriers to help seeking in teenage and young adults (TYA). Presentation at the National Proms Conference Birmingham. 2018.

[10] Holch P, Jones G, Vaughan K, McCabe MG. Cancer awareness, risk perception, self-efficacy and barriers to help seeking in teenage and young adults (TYA). Presentation at the International Psychosocial Oncology Society Congress Hong Kong Psycho-Oncol. 2018;27(S3). 10.1002/pon.4878.

[11] Morley H, Holch P, Vaughan K, McCabe MG. Cancer awareness and the barriers to help seeking behaviour in young people: a cognitive interview study. Presentation at the British Psychosocial Oncology Society. Psycho-Oncol. 2018;27(S2):19-20.

[12] Musavian AS, Pasha A, Rahebi SM, et al. Health promoting behaviors among adolescents: a cross-sectional study. Nurs Midwifery Stud. 2014;3(1):e14560. 10.17795/nmsjournal14560. 25414892 10.17795/nmsjournal14560 PMC4228521

[13] Gibson AM, Shaw J, Hewitt A, Easton C, Robertson S, Gibson N. A longitudinal examination of students’ health behaviours during their first year at university. J Furth High Educ. 2018;42(1):36–45. 10.1080/0309877X.2016.1188902.

[14] Brown MC, Araújo-Soares V, Skinner R, Glaser AW, Sarwar N, Saxton J, et al. Using qualitative and co-design methods to inform the development of an intervention to support and improve physical activity in childhood cancer survivors: a study protocol for BEing Active after ChildhOod caNcer (BEACON). BMJ Open. 2020;10(12):e041073. 33371034 10.1136/bmjopen-2020-041073 PMC7754664

[15] Stapley S, Hamilton W. Gynaecological symptoms reported by young women: examining the potential for earlier diagnosis of cervical cancer. Fam Pract. 2011;28(6):592–8. 10.1093/fampra/cmr033. 21632969 10.1093/fampra/cmr033

[16] Ebell MH, Culp MB, Radke TJ. A systematic review of symptoms for the diagnosis of ovarian cancer. Am J Prev Med. 2016;50(3):384–94. 10.1016/j.amepre.2015.09.023. 26541098 10.1016/j.amepre.2015.09.023

[17] Browne JL, Chan AY. Mother-daughter communication about mammography in an Australian sample. J Fam Commun. 2012;12(2):129–50.

[18] Mosavel M. The feasibility of mothers accepting health advice from their adolescent daughters. J Health Care Poor Underserved. 2009;20(1):42–9. 19202244 10.1353/hpu.0.0102

[19] Smith S, Case L, Fern L, Waterhouse K, Vaughan J, McCabe M. Poor cancer awareness in young people in Greater Manchester advocates the need for age-specific interventions to raise awareness. In: Cancer Conference. Liverpool, UK: NCRI Cancer Conference. 2016.

[20] Walsh J, O’Reilly M, Treacy F. Factors affecting attendance for a cervical smear test: a prospective study. 2003. https://www.cervicalcheck.ie/_fileupload/Publications/Factors%20affecting%20attendance%20at%20smear%20tests%20Sept%2003.pdf. Accessed 19 Jan 2024.

[21] Skivington K, Matthews L, Simpson SA, Craig P, Baird J, Blazeby JM, et al. A new framework for developing and evaluating complex interventions: update of Medical Research Council guidance. BMJ (Clinical research ed). 2021;374:n2061. 10.1136/bmj.n2061. 34593508 10.1136/bmj.n2061 PMC8482308

[22] Lancaster GA. Pilot and feasibility studies come of age! Pilot Feasibility Stud. 2015;1(1):1. 29611687 10.1186/2055-5784-1-1 PMC5842886

[23] Lewis M, Bromley K, Joseph R. Small numbers, big decisions: guidance for sample size in pilot trials. BMJ. 2025;390:10. 10.1136/bmj.r1885 40930812

[24] Teresi JA, Yu X, Stewart AL, Hays RD. Guidelines for designing and evaluating feasibility pilot studies. Med Care. 2022;60(1):95–103. 10.1097/MLR.0000000000001664. 34812790 10.1097/MLR.0000000000001664 PMC8849521

[25] Orbell S, Hodgkins S, Sheeran P. Implementation intentions and the theory of planned behavior. Pers Soc Psychol Bull. 1997;23(9):945–54. 10.1177/0146167297239004. 29506445 10.1177/0146167297239004

[26] Wilding S, Tsipa A, Branley-Bell D, Greenwood DC, et al. Cluster randomized controlled trial of volitional and motivational interventions to improve bowel cancer screening uptake: a population-level. Soc Sci Med. 2020;265:113496. 33189426 10.1016/j.socscimed.2020.113496

[27] Wilding S, Wighton S, West R, Conner M, O’Connor DB. A randomised controlled trial of volitional and motivational interventions to improve cervical cancer screening uptake. Social science & medicine (1982). 2023;322:115800. 10.1016/j.socscimed.2023.115800. 36858020 10.1016/j.socscimed.2023.115800

[28] Stubbings S, Robb K, Waller J, Ramirez A, Austoker J, Macleod U, et al. Development of a measurement tool to assess public awareness of cancer. Br J Cancer. 2009;101(Suppl 2):S13–7. 10.1038/sj.bjc.6605385. 19956157 10.1038/sj.bjc.6605385 PMC2790699

[29] Smith S, Case L, Fern L, Waterhouse K, Vaughan K, McCabe M. Poor cancer awareness in young people in Greater Manchester advocates the need for age-specific interventions to raise awareness. In: NCRI Cancer Conference, 6–9 November, Liverpool, UK. 2011.

[30] Ajzen I. The theory of planned behavior. Organ Behav Hum Decis Process. 1991;50(2):179–211.

[31] Conner M, Armitage CJ. Extending the theory of planned behavior: a review and avenues for further research. J Appl Soc Psychol. 1998;28(15):1429–64.

[32] Mowbray FI, Manlongat D, Shukla M. Sensitivity analysis: a method to promote certainty and transparency in nursing and health research. Can J Nurs Res = Revue canadienne de recherche en sciences infirmieres. 2022;54(4):371–6. 10.1177/08445621221107108 PMC9605992 35702010

[33] Tabachnick BG, Fidell LS. Using multivariate statistics. 6th ed. Boston, MA2013: Allyn and Bacon; 2103.

[34] Eldridge SM, Chan CL, Campbell MJ, et al. CONSORT 2010 statement: extension to randomised pilot and feasibility trials. BMJ. 2016;355:i5239. 10.1136/bmj.i5239. 27777223 10.1136/bmj.i5239 PMC5076380

[35] Braun V, Clarke V. Using thematic analysis in psychology. Qual Res Psychol. 2006;3(2):66–106.

[36] Braun V, Clarke V. Using thematic analysis in psychology. Qual Res Psychol. 2006;3(2):77–101. 10.1191/1478088706qp063oa.

[37] Braun V, Clarke V. Reflecting on reflexive thematic analysis. Qual Res Sport Exerc Health. 2019;11(4):589–97.

[38] Clarke V, Braun V. Thematic analysis: a practical guide. Thematic analysis. 2021:1–100.

[39] Mellor K, Albury C, Dutton SJ, et al. Recommendations for progression criteria during external randomised pilot trial design, conduct, analysis and reporting. Pilot Feasibility Stud. 2023;9:59. 37061720 10.1186/s40814-023-01291-5 PMC10105402

