# Supplementary material for: Study protocol, increasing awareness and early detection in students at risk of gynaecological cancer: feasibility of an online educational and behaviour change intervention
Source: Pilot Feasibility Stud. 2026 Apr 21;12:83. doi: 10.1186/s40814-026-01801-1 (PMC13227870; doi:10.1186/s40814-026-01801-1)
Supplement: Supplementary file 1 — Supplementary Material 1. [file 40814_2026_1801_MOESM1_ESM.docx]

**Supplementary item 1**

**Young People’s Cancer Awareness Measure (YPCAM)**


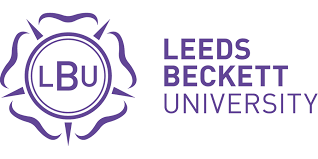


The original survey instrument (CAM) on which this was based was developed by Cancer Research UK, University College London, Kings College London, and University of Oxford in 2007-2008.

The YPCAM has been adapted by members of the YPCAM steering group in combination with the Teenage Cancer Trust and the University of Manchester

It has further been refined in 2020 at Leeds Beckett University using cognitive interviews from with a sample of young people 16-25


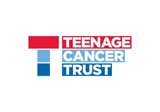

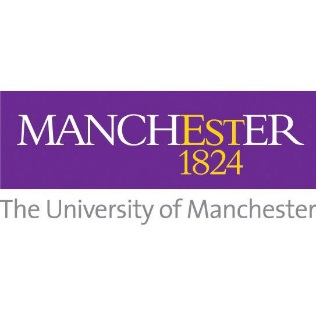


**The questions in this questionnaire are only about cancers that happen in young people (teenagers and young adults under 30 years of age).**

**The questionnaire is not a test. Your answers will not be marked right or wrong. We are interested in finding out how much you actually know about cancer rather than how much you think you should know about cancer.**

| **Q2. Cancer in young people is rare. However, which of the following do you think could be warning signs of cancer in young people?**  Yes No Don’t know |
| --- |
| Do you think an unexplained or unexpected lump  or swelling could be a sign of cancer?  Do you think persistent unexplained pain (that  doesn’t go away or keeps coming back)  (including headache) could be a sign of cancer?  Do you think unexpected bleeding e.g. in urine/poo/vomit) after sex or between periods could be a sign of cancer?  Do you think unexplained fatigue (extreme tiredness) could be a sign of cancer?  Do you think frequent, drenching sweats at night  could be a sign of cancer?  Do you think persistent/unexplained (that doesn’t  go away or keeps coming back) vomiting could be a sign of cancer?  Do you think a change in the appearance of a  mole could be a sign of cancer?  Do you think unexpected weight loss could be a  sign of cancer?  Do you think unexplained weight gain could be a  sign of cancer ?  Do you think unexplained bruising could be a  sign of cancer?  Do you think regularly getting out of breath could be a  A sign of cancer?  Do you think persistent constipation or diarrhoea could  be a sign of cancer?  Do you think persistent dizziness could be  a sign of cancer ? |

**Please answer the following questions in the order they appear without looking for answers in later questions.**

**Q1. There are many warning signs (symptoms) of cancer in young people.**

**Please name as many as you can think of:**

| **Q3. What do you think are the three most common cancers in young people?** |
| --- |
| 1  2  3 |

| **Q4. Which of t**  **Which of these cancers do you think young people get? please tick all that apply**  Breast cancer | Brain tumour | **hese cancers these do you think young people can get?**  Testicular  Leukaemia Sarcoma  cancer | Melanoma  (skin cancer) | Bowel cancer |
| --- | --- | --- | --- | --- |
|  |  |  |  |  |
| Lymphoma  (cancer of the lymph glands) | Cervical cancer | Throat  Liver cancer Bone cancer  cancer | Cancer of the ovaries | Lung cancer |
|  |  |  |  |  |

| **Q5. If you noticed the following things how soon would you make an appointment with your doctor to discuss them?**  **Within Within Within Within Within Within**  **Never**  **1 week 2 weeks 1 month 3 months 6 months 12 months** |
| --- |
| An unexplained lump or  swelling  Unexpected pain /  headache  Unexplained night sweats  Persistent or unexplained  vomiting  Fatigue (extreme tiredness)  A change in the  appearance of a mole  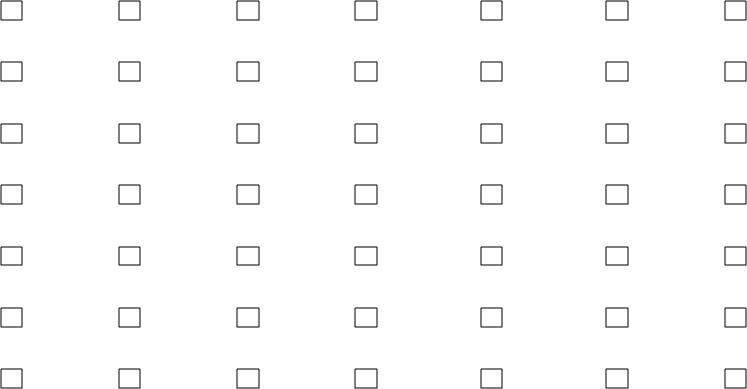Unexplained weight loss  Unexplained bruising  Bleeding you can’t explain  (e.g. in urine/poo/vomit)  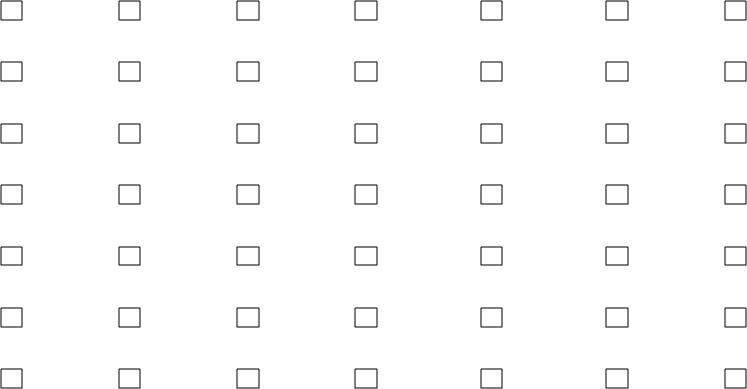after sex or between periods  Ongoing changes when you go  for a poo (e.g. constipation or  diarrhoea (or both), pain, or  feeling like you’ve  not quite finished going  Getting out of breath more  easily than normal  Persistent dizziness  Another symptom you think  may be cancer |

| **Q6. Sometimes people don’t go to see the doctor, even when they have a symptom that they think might be serious. These are some of the reasons young people give for this. Could you say if any of these might put you off going to the doctor?**  Yes Possibly No Don’t know |
| --- |
| I would be too embarrassed  I would be too scared to embark on the process  I would be worried about wasting the doctor’s time  My doctor would be difficult to talk to  It would be difficult to make an appointment with my doctor  I would be too busy to make time to go to the doctor  I would be too worried about taking time off from school,  college or work  I have too many other things to worry about  It would be difficult for me to get to the doctor’s surgery  I would be worried about what the doctor might find  I would find it hard to talk to my doctor  I don’t like my doctor  My doctor is the wrong gender  Other (please say what): |

| **Q7. If you had a sign or symptom that you were worried about and had told your doctor but thought that she/he/they hadn’t taken it seriously, would you:**  Yes Possibly No Don’t know |
| --- |
| Try to ignore the symptom  Try to see another doctor  Ask someone else to go with you to the doctor, like your  parents or partner  Talk to someone else about it, like a tutor or  friend  Look it up on the internet  Go to the hospital A&E department about it  Do something else (please specify what) |

| **Q8. If you had a sign or symptom that you were worried about and had told someone that you trust such as your parents or partner but you thought that they hadn’t taken it seriously, would you:**  Yes Possibly No Don’t know |
| --- |
| Try to ignore the symptom  Go to see your doctor  Talk to someone else about it, like a tutor or  friend  Look it up on the internet  Go to the hospital A&E department about it  Do something else (please say what):      6 |

| **Q9. How many young people do you think find out they have cancer each day in the UK?**  None 1-10 11-20 21-50 51-100 More than 100 |
| --- |
|  |

| **Q10. These are some of the things that can increase your chance of developing cancer later in life. How much do you agree that each may increase your chance of developing cancer as a young person?**    Strongly disagree Disagree Don’t know Strongly agree Strongly agree |
| --- |
| Smoking any cigarettes at all  Breathing in another person’s cigarette smoke  (passive smoking)  Drinking more than 1 unit of alcohol a day  (small glass of wine, ‘shot’ of spirits, 1/2 pint of beer)  Eating fewer than 5 portions of fruit and vegetables a day  Eating red or processed meat (like ham, bacon, salami) once a day or more  Being overweight (BMI over 25)  Getting sunburnt more than once as a child  Having a close relative with cancer  Infection with HPV (Human Papillomavirus)  Doing less than 30 mins of moderate physical  activity (such as walking to school or work,  walking the dog) 5 times a week  Using a sunbed  Please list any other things you can think of that affect your chances of getting cancer as a young person:  Insert text box |

**Young People’s Cancer Awareness Measure (YPCAM)**

**Demographic Questions**

We would now like to ask you a few questions about yourself to help to analyse the study. You will not be asked your name and your answers will be kept private and confidential.

| **1. What is your age?** |  |  |  |
| --- | --- | --- | --- |
|  |  | |  |
|  |  | |  |
| **2. Can you describe your gender** |  | |  |
|  |  | |  |

| **3. Which of these best describes your ethnic group?** | | |  |  |
| --- | --- | --- | --- | --- |
| **White** | **Mixed** | **Asian or Asian British** | **Black or Black British** | **Chinese/other** |
| White British | White and  Black  Caribbean | Indian | Black  Caribbean | Chinese |
| White Irish | White and  Black African | Pakistani | Black African | Other……… |
| Any other  White background | White and Asian | Bangladeshi | Any other Black background |  |
|  | Any other Mixed background | Any other Asian background |  |  |

| **4. What is your marital status?** |  |  |  |  |  |
| --- | --- | --- | --- | --- | --- |
| Single/never Married/living married with partner | Married separated from | Divorced |  | Widowed | Civil partnership |
| Prefer not to say |  |  |  |  |  |
|  |  |  |  |  |  |

| **5. Please tick the box which best describes your living arrangement:** |  |
| --- | --- |
| Live at home Other (e.g. living University Live with Partner with parents with family/friends) Accommodation | Live Alone |

**6. What is your Home town (i.e. not student residence) Postcode (please limit to the first 4 digits)?**

| **7. Are you currently:** |  |  |
| --- | --- | --- |
|  |  | Unemployed |
|  |  | Employed full-time |
| University student (which university?) |  | Employed part-time |
| Self-employed |  | Disabled or too ill to work |

| **8. What is the highest level of education qualification you have obtained** |
| --- |
| Degree or higher degree GCSE equivalent (Grade A-C)  A-levels, Highers or equivalent GCSE equivalent (Grade D-G)  BTEC/GNVQ No formal qualifications  Still at school Prefer not to say |

| **9. Have you, your family or close friends had cancer? (please tick all that apply)** | | | | |  |  |
| --- | --- | --- | --- | --- | --- | --- |
| You | Yes | No | Close Friend | Yes |  | No |
| Partner | Yes | No | Other Friend | Yes |  | No |
| Close family member | Yes | No | Not sure |  |  |  |
| Other family member | Yes | No | Do not wish to answer |  |  |  |

| **10. Are you registered with a GP?** |  |  |  |
| --- | --- | --- | --- |
| Yes |  |  | No |

| **11. What is the main language spoken at home?** |
| --- |
| English Sylheti  Urdu Cantonese  Punjabi Other…………………..  Gujarati |

**Supplementary information 2**

**Theory of Planned Behaviour - Survey Questions**

**Attitudes**

Gaining knowledge about different gynaecological cancers and their symptoms is important:

agree :___1__:___2__:___3__:___4__:___5__:___6__:___7___: disagree

I think monitoring for gynaecological cancers is worth doing:

agree :___1__:___2__:___3__:___4__:___5__:___6__:___7___: disagree

It is embarrassing to talk about symptoms relating to gynaecological cancers:

agree :___1__:___2__:___3__:___4__:___5__:___6__:___7___: disagree

Getting symptoms checked is painful (i.e., pap smear, breast examination):

agree :___1__:___2__:___3__:___4__:___5__:___6__:___7___: disagree

Getting symptoms checked is embarrassing:

agree :___1__:___2__:___3__:___4__:___5__:___6__:___7___: disagree

**Anticipated regret**

I would feel regret if I didn’t take the time to learn about the signs and symptoms of gynaecological cancer:

agree :___1__:___2__:___3__:___4__:___5__:___6__:___7___: disagree

I would feel regret if I didn’t check myself for any of these symptoms after learning about them:

agree :___1__:___2__:___3__:___4__:___5__:___6__:___7___: disagree

I would feel regret if I didn’t go to the doctor with symptoms relating to gynaecological cancer:

agree :___1__:___2__:___3__:___4__:___5__:___6__:___7___: disagree

**Perceived norms**

Most people who are important to me approve of my learning about different gynaecological cancers and their symptoms.

agree :___1__:___2__:___3__:___4__:___5__:___6__:___7___: disagree

My family think I should monitor myself for gynaecological cancers:

agree :___1__:___2__:___3__:___4__:___5__:___6__:___7___: disagree

My closest friends think I should monitor myself for gynaecological cancers:

agree :___1__:___2__:___3__:___4__:___5__:___6__:___7___: disagree

Most people like me (similar age/sex) are aware of the different gynaecological cancers and their symptoms:

agree :___1__:___2__:___3__:___4__:___5__:___6__:___7___: disagree

**Perceived behavioural control**

I am confident that I can check myself for gynaecological cancer symptoms:

agree :___1__:___2__:___3__:___4__:___5__:___6__:___7___: disagree

My awareness of my body and possible changes that happen is up to me to keep on top of:

agree :___1__:___2__:___3__:___4__:___5__:___6__:___7___: disagree

**Behavioural Intentions**

I am willing to check myself once a month for any physical gynaecological cancer symptoms:

agree :___1__:___2__:___3__:___4__:___5__:___6__:___7___: disagree

I am willing to go to the doctor if I find any of the symptoms of gynaecological cancer:

agree :___1__:___2__:___3__:___4__:___5__:___6__:___7___: disagree

I am willing to talk to friends about gynaecological cancers and its symptoms:

agree :___1__:___2__:___3__:___4__:___5__:___6__:___7___: disagree

**Self-efficacy**

I believe I can solve any difficulties faced by self-monitoring:

agree :___1__:___2__:___3__:___4__:___5__:___6__:___7___: disagree

If I know that self-monitoring has many benefits, even if others object, I will still do it:

agree :___1__:___2__:___3__:___4__:___5__:___6__:___7___: disagree

It is easy for me to insist on having symptoms checked by a doctor:

agree :___1__:___2__:___3__:___4__:___5__:___6__:___7___: disagree

If I have gynaecological cancer, I believe I can effectively cope with the resulting problems:

agree :___1__:___2__:___3__:___4__:___5__:___6__:___7___: disagree

If I have gynaecological cancer, I can calmly face it because I believe in my ability to handle problems:

agree :___1__:___2__:___3__:___4__:___5__:___6__:___7___: disagree

**Supplementary item 3**

**Semi-Structured Interview schedule**

Welcome, introduction and pre-amble about the project –right to withdraw, confidentiality and anonymity

**Introduction:**

Hi, my name is [the interviewer’s name]. I would like to thank you for sparing the time to discuss your experience of taking part in this research. Give information and consent form. Make sure understand everything, ask any questions want to ask.

**Questions:**

**Views on the educational film**

- How do you feel about the information shown in the short film?
- How do you feel about what you learned from short film?
- How did you find the length of the video?
- How did you feel while watching it/what emotions did you feel?
- Do you feel like you understood everything in the short video?
- How did you find the language used in the video?

**Questionnaires**

- What was completing the questionnaire like?
- How was the length of the questionnaire?

**Volitional help sheet**

- What was filling in the help sheet like?

**Practical issues**

- How was your experience of accessing everything online?

**Changes you have made as a result of the intervention**

- How has the intervention changed your cancer awareness?
- Describe how your behaviours might change after this intervention. (More checking monitoring/future attendance to screening)
- Describe how your outlook on gynaecological cancers has changed.
- How will you talk to other family and friends about this experience?
- What was the most effective part of the film/volitional help sheet etc?
- How could we improve the study/intervention?
- Do you have anything else to add?

Thank you and debrief
